# Regulation of Fibroblast Growth Factor-2 Expression and Cell Cycle Progression by an Endogenous Antisense RNA

**DOI:** 10.3390/genes3030505

**Published:** 2012-08-16

**Authors:** Mark Baguma-Nibasheka, Leigh Ann MacFarlane, Paul R. Murphy

**Affiliations:** Department of Physiology and Biophysics, Faculty of Medicine, Dalhousie University, 5850 College Street, Halifax, NS B3H 4R2, Canada; E-Mails: mbaguma@yahoo.com (M.B.-N.); alexandl@dal.ca (L.A.M.)

**Keywords:** antisense RNA, FGF2, NUDT6, siRNA, cell cycle

## Abstract

Basic fibroblast growth factor (FGF2) is a potent wide-spectrum mitogen whose overexpression is associated with immortalization and unregulated cell proliferation in many tumors. The FGF2 gene locus is bi-directionally transcribed to produce FGF2 mRNA from the “sense” strand and a *cis*-antisense RNA (NUDT6) from the NUDT6 gene on the “antisense” strand. The NUDT6 gene encodes a nudix motif protein of unknown function, while its mRNA has been implicated in the post-transcriptional regulation of FGF2 expression. FGF2 and NUDT6 are co-expressed in rat C6 glioma cells, and ectopic overexpression of NUDT6 suppresses cellular FGF2 accumulation and cell cycle progression. However, the role of the endogenous antisense RNA in regulation of FGF2 is unclear. In the present study, we employed siRNA-mediated gene knockdown to examine the role of the endogenous NUDT6 RNA in regulation of FGF2 expression and cell cycle progression. Knockdown of either FGF2 or NUDT6 mRNA was accompanied by a significant (>3 fold) increase in the complementary partner RNA. Similar reciprocal effects were observed at the protein level, indicating that these two transcripts are mutually regulatory. Remarkably, knockdown of either FGF2 or NUDT6 significantly reduced cell proliferation and inhibited S-phase re-entry following serum deprivation, implicating both FGF2 and NUDT6 in the regulation of cell transformation and cell cycle progression.

## 1. Introduction

Whole-genome sequencing reveals that approximately 20% of the human genome is bi-directionally transcribed, and an increasing number of endogenous “antisense” transcripts have been identified [1], which may play a role in the transcriptional and post-transcriptional regulation of the cognate gene products [2,3]. However, the physiological significance of these antisense transcripts and their role(s), if any, in the regulation of sense gene expression remain poorly understood. Perhaps one of the best-defined examples of bi-directional transcription involves the basic fibroblast growth factor (FGF2) gene and its antisense partner, NUDT6 (also known as FGF-AS or GFG). Basic fibroblast growth factor (FGF2, encoded on the “sense” DNA strand), is a pleiotropic factor implicated in a multitude of physiologic and pathologic processes, including angiogenesis, wound healing, and tumor growth. The antisense transcript encodes NUDT6, a member of the conserved nudix-motif family of “housekeeping” enzymes [4] which has also been implicated in the regulation of cell proliferation [5,6,7]. However, the NUDT6 mRNA is fully complementary to the FGF2 mRNA 3’UTR over a span of more than 400 nucleotides [8,9], and several lines of evidence reviewed below support the hypothesis that NUDT6 mRNA functions as regulatory antisense RNA in the post-transcriptional control of FGF2 expression.

The sense-antisense organization of the FGF2/NUDT6 gene locus is highly conserved across vertebrate species, suggesting an essential functional relationship [8,9,10,11]. The expression and translation of FGF2 and NUDT6 are tightly linked, and show reciprocal patterns of expression in a variety of tissues during development [12,13], and in normal *vs*. tumor cells [5,14,15,16]. The relative expression of FGF2 and NUDT6 vary dynamically and reciprocally through the cell cycle [14,17], and disruption of the relative expression of FGF2 and its antisense transcript has been associated with increased tumor progression in esophageal adenocarcinoma [15,16] and pituitary adenomas [5] and increased invasiveness of endometrial tissue in patients with endometriosis [18]. We have previously demonstrated that constitutive or Tet-inducible over-expression of recombinant NUDT6 inhibits cell cycle progression and proliferation in rat [5,19,20] and human [16] tumor cells. However, there is conflicting data regarding whether these effects are directly attributable to antisense RNA-mediated knockdown of FGF2 expression, or to effects of the encoded NUDT6 protein on cell proliferation. In SEG-1 cells and C6 glioma cells NUDT6 over-expression reduced cellular FGF2 immunoreactivity and inhibited cell cycle progression and proliferation [16,19], whereas in rat GH3 pituitary adenoma cells NUDT6 inhibited cell cycle progression and proliferation, but did not suppress FGF2 levels [5]. More recently, overexpression of recombinant NUDT6 in human colorectal cancer cells was reported to increase cell proliferation and colony formation in soft agar, raising the possibility that NUDT6 protein itself has proliferative and/or oncogenic activity [7]. These contradictory results may reflect species- or cell-specific differences, or differences in transfection protocols or site of integration. In any event, they do not address the possible role of endogenous NUDT6 in regulating FGF2. 

In the present report, we have used an siRNA-mediated gene knockdown approach to further elucidate the role of endogenous NUDT6 in the regulation of FGF2 expression and function in rat C6 glioma cells. 

## 2. Results and Discussion

### 2.1. Reciprocal Effects of FGF2 and NUDT6 Knockdown on mRNA Levels

**Figure 1:**
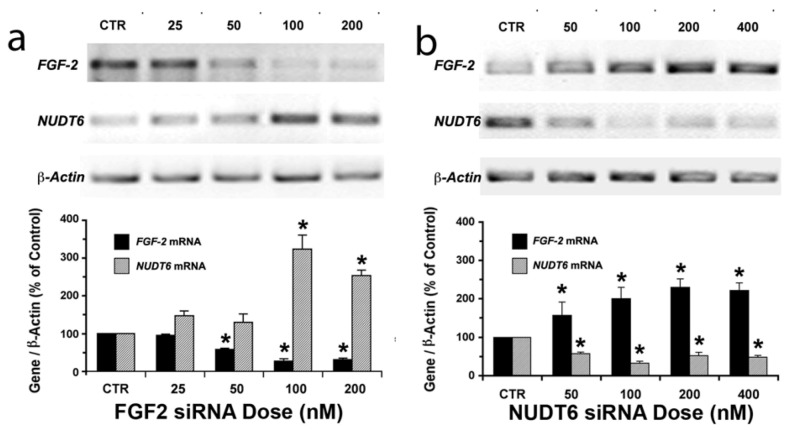
Dose-dependent reciprocal effects of siRNA knockdown on FGF2 and NUDT6. (**a**) FGF2 knockdown up-regulates the antisense (NUDT6) mRNA; and (**b**) NUDT6 knockdown up-regulates FGF2 mRNA. C6 glioma cells were transfected with the indicated doses of siRNA or 100 nM negative control (CTR) siRNA. Total RNA was isolated after 48 hours for RT-PCR. Expression intensity was normalized against β-actin amplified from the same RT reaction. * significantly different from control, *p* < 0.05, n = 3 independent transfections.

**Figure 2:**
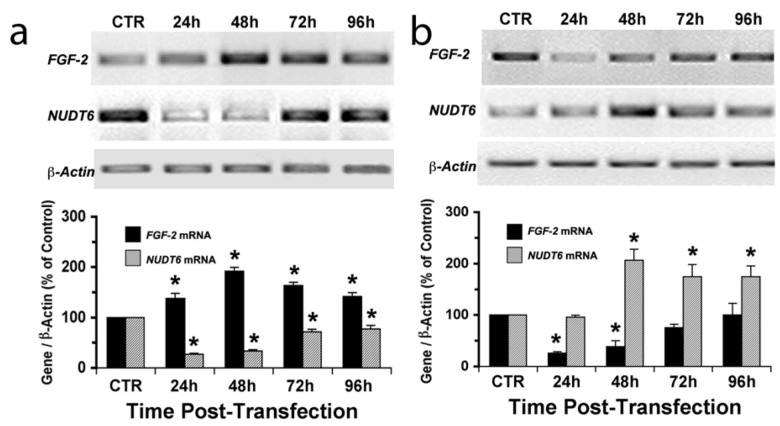
Time course of siRNA knockdown of FGF2 and NUDT6 mRNA. C6 glioma cells were transfected with 100 nM of NUDT6 siRNA (**a**), FGF2 siRNA (**b**), or a negative control siRNA (CTR). Total RNA was isolated at the indicated times (48 hours for the CTR group), and the expression intensity was normalized against β-actin amplified from the same RT reaction. * significantly different from CTR, *p* < 0.05, n = 3.

To achieve successful silencing of *FGF2* and *NUDT6*, we initially compared the effects of three Silencer^TM^ Select Pre-designed siRNA variants on FGF2 and NUDT6 mRNA expression. In each case, all three tested sequences significantly decreased the targeted mRNA (*p* < 0.05, data not shown). FGF2 siRNA variant *s132467* and NUDT6 siRNA variant *s220427* had the greatest effect on their respective target mRNAs, and were therefore chosen for use in all subsequent experiments. The mRNA knockdown was dose- and time-dependent (Figure 1 and Figure 2). Significant knockdown of either target was evident within 48 hours following treatment with as little as 50 nM siRNA. As shown in Figure 1a, FGF2 knockdown with 100 or 200 nM siRNA (but not lower doses) resulted in a significant (3-fold) increase in NUDT6 mRNA. In contrast, even the lowest dose (50 nM) of NUDT6 siRNA significantly increased FGF2 mRNA expression, in conjunction with NUDT6 knockdown (Figure 1b). 

The effects of siRNA knockdown of the target genes were detectable as early as 24 hours and persisted for up to 96 hours (Figure 2). The up-regulation of FGF2 following NUDT6 knockdown was evident within 24 hours, and remained elevated for the 96-hour duration of the experiment (Figure 2a). NUDT6 mRNA was also up-regulated following FGF2 silencing, although the response was slower in onset, not being detectable until 48 hours after siRNA treatment. Interestingly, NUDT6 mRNA remained elevated at 72 and 96 hours following FGF2 siRNA treatment, even after FGF2 mRNA levels had returned to normal (Figure 2b). Taken together these data strongly support the hypothesis that FGF2 and NUDT6 mRNAs are mutually regulatory. We next examined the effect of the siRNA knockdowns on the levels of the encoded proteins.

### 2.2. Effect of NUDT6 Knockdown on FGF2 Protein Isoform Expression

**Figure 3:**
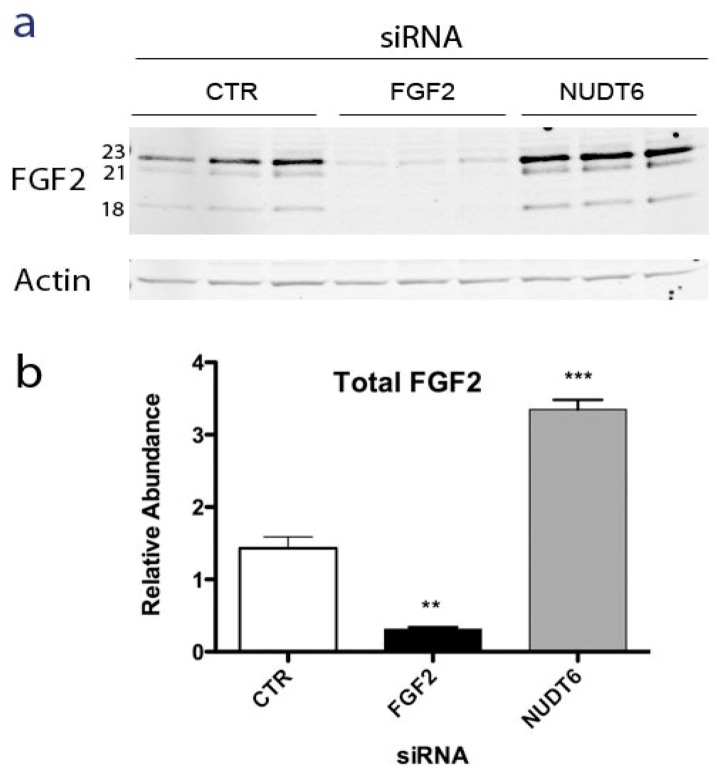
(**a**) Representative Western blot of FGF2 at 72 hours after transfection with 100 nM of a negative control siRNA (CTR), FGF2 siRNA, or NUDT6 siRNA. Numbers indicate the molecular weights of the three major FGF2 isoforms. Actin immunostaining was used as a loading control; (**b**) Densitometric analysis of total FGF2 immunoreactivity. Results are the average ± SEM of three independent transfections. **, *p* < 0.01 *vs*. CTR; ***, *p* < 0.001 *vs*. CTR.

Alternative translation initiation results in synthesis of five isoforms of FGF2 ranging from 18 to 34 kDa in size, with distinct intracellular localizations and actions [21,22,23]. The low molecular weight (LMW) 18kDa FGF2 isoform is secreted by a non-classical pathway [24] and binds to high affinity cell surface receptors [25]. The high molecular weight (HMW) FGF2 isoforms contain nuclear and nucleolar localization signals and have cell survival, proliferation and immortalization activities [26,27]. In order to determine if NUDT6 knockdown differentially affected the expression of the various FGF2 isoforms, Western blot analysis was used to determine the relative abundance of LMW and HMW FGF2 following siRNA knockdown of either FGF2 or NUDT6. As shown in Figure 3a, C6 glioma cells predominantly express three FGF2 isoforms; the LMW 18 kDa isoform and two HMW isoforms of 21 and 23 kDa, with the 23 KDa isoform being most abundant. 

FGF2 knockdown significantly attenuated all three isoforms, whereas NUDT6 knockdown resulted in up-regulation of all FGF2 isoforms. Densitometric analysis did not detect any significant differential effect on individual isoforms (not shown). As depicted in Figure 3b, total FGF2 immunoreactivity was significantly up-regulated more than 3-fold following NUDT6 knockdown, resulting from comparable up-regulation of each of the three major FGF2 isoforms. 

### 2.3. Effect of RNAi-Mediated Knockdown of FGF2 and NUDT6 mRNA on Their Cognate Proteins

**Figure 4:**
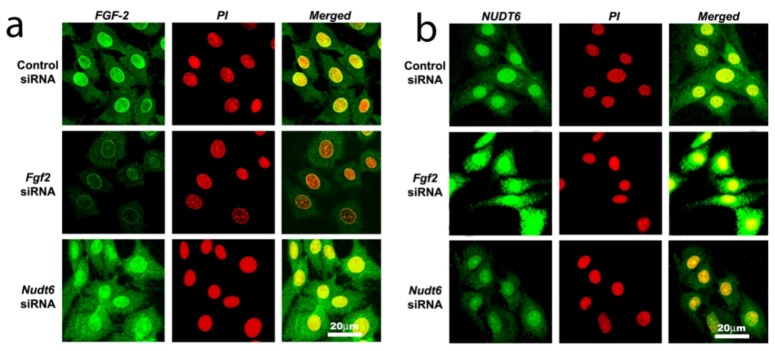
Confocal immunofluorescence detection of FGF2 protein (green in Panel **a**) and NUDT6 (green in Panel **b**) in C6 glioma cells 72 hours after transfection with100 nM of a control negative scramble sequence, FGF*2* siRNA or NUDT6 siRNA. Red indicates propidium iodide (PI) staining of cell nuclei.

Confocal laser scanning microscopy (CLSM) analysis confirmed that immunoreactive FGF2 protein was present in the cytoplasmic, perinuclear and nuclear compartments in unsynchronized cells transfected with scrambled control siRNA (Figure 4a, top panels). In contrast, NUDT6 immunoreactivity showed significant nuclear staining, as well as punctate extra-nuclear staining consistent with the mitochondrial localization previously described [28] for rat NUDT6 (Figure 4b, top panel). Following 72 hours of FGF2 siRNA transfection, FGF2 immunostaining in both nucleus and cytoplasm was markedly attenuated, whereas it was markedly up-regulated in both compartments following NUDT6 knockdown (Figure 4a, middle and bottom panels). Conversely, NUDT6 immunostaining was dramatically up-regulated following *FGF2* siRNA treatment, and greatly attenuated by NUDT6 knockdown (Figure 4b, middle and bottom panels). These data indicate that the FGF2 and NUDT6 protein levels are mutually regulated by their antisense RNA partner in C6 glioma cells.

**Figure 5:**
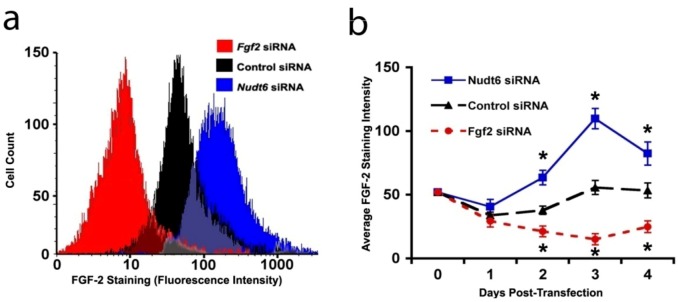
(**a**) Representative immunofluorescence histograms of FGF2 staining 72 hours following transfection with 100 nM siRNA; (**b**) Average FGF2 staining intensity over the course of four days after the indicated transfections. Asterisks indicate that each treatment was different from the other two at the same time points. *p* < 0.05, n = 3.

In order to confirm that the changes in FGF2 protein expression detected by CLSM reflected actual alterations in cellular protein content, we performed a fluorescence-activated cell sorting (FACS) analysis of FGF2 content in the C6 cells following siRNA mediated knockdown of FGF2 or NUDT6. As shown in Figure 5a, FGF2 siRNA significantly reduced the total cellular FGF2 content (as indicated by the mean staining intensity) 72 hours following siRNA transfection, whereas transfection with the NUDT6 siRNA significantly increased the FGF2 levels. Furthermore, as shown in Figure 5b, the up-regulation of cellular FGF2 immunoreactivity following NUDT6 knockdown was detectable within 48 hours, and the effect persisted for the duration of the experiment. Similarly, FGF2 siRNA transfection significantly suppressed FGF2 within 48 hours following transfection, and the inhibition persisted for up to 4 days following transfection (Figure 5b). 

### 2.4. Effect of FGF2 and NUDT6 Knockdown on Cell Proliferation

**Figure 6:**
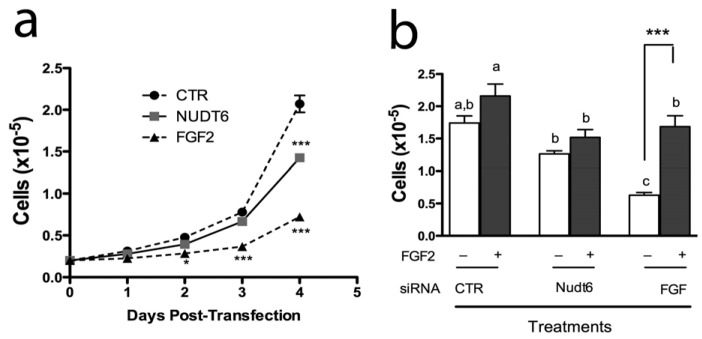
(**a**) Effect of siRNA knockdown on proliferation of C6 glioma cells. Cells were counted on successive days following transfection with 100nM of the indicated siRNAs. *, *p* < 0.05 *vs*. control; ***, *p* < 0.001 *vs*. control; n = 3; (**b**) Recombinant FGF2 reverses the inhibition of cell proliferation resulting from FGF2 siRNA treatment. Cells were transfected with gene-specific siRNAs (solid bars) or negative control siRNA (open bars) with (+) or without (–) addition of 10 ng/mL recombinant FGF2. Groups with different letters are significantly different from each other.

We have previously reported that endogenous FGF2 is required for C6 glioma cell proliferation and that FGF2 antisense oligonucleotides inhibit C6 cell proliferation and colony formation in soft agar [29]. However, the physiological significance of the NUDT6 gene for FGF regulation of cell proliferation has not been fully clarified. We therefore examined the effect of FGF2 and NUDT6 knockdown on C6 glioma cell growth. As shown in Figure 6a, knockdown of FGF2 significantly inhibited cell proliferation, detectable within 48 hours post-transfection. This inhibition persisted for the duration of the experiment, becoming highly significant at 96 hours post-transfection (0.72 ± 0.08 × 10^5^ *vs*. 2.07 ± 0.18 × 10^5^ for the negative control transfected cells). Remarkably, knockdown of NUDT6 also resulted in inhibition of cell proliferation, although this did not reach significance until 4 days post-transfection, and achieved only 31% inhibition *vs*. control, compared to 65% inhibition seen following FGF2 knockdown. 

In order to determine if the inhibition of cell proliferation was a direct result of perturbation of FGF2 expression, we examined the effect of exogenous FGF2 addition to siRNA transfected cells. As shown in Figure 6b, recombinant FGF2 caused a modest but non-significant increase in proliferation in cells transfected with a negative control siRNA. In contrast, FGF2 siRNA transfection caused a highly significantly 64% inhibition of cell proliferation, and this effect was completely reversed by addition of 10 ng/mL FGF2 to the culture medium. In contrast, addition of exogenous FGF2 had no significant effect on proliferation of cells transfected with NUDT6 siRNA.

### 2.5. Effect of Knockdown on S-phase Entry and Cell Cycle Progression

**Figure 7:**
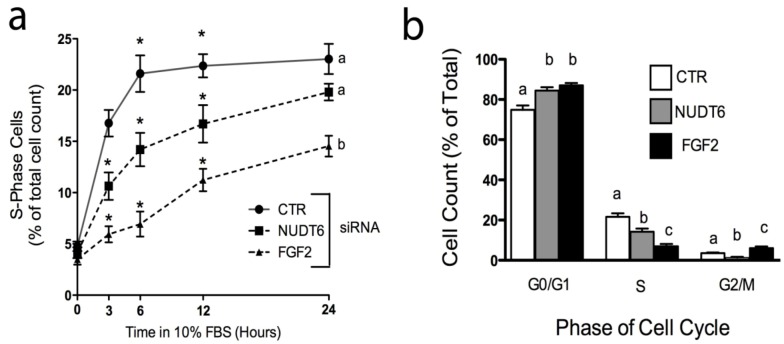
Effect of FGF2 and NUDT6 knockdown on S-phase entry and cell cycle progression. (**a**) The percentage of cells in S-phase at the indicated times following return to 10% FBS. *, *p* < 0.05 *vs*. other treatments at each time point. Groups with different letters are significantly different from each other, *p* < 0.05, n = 3; (**b**) Cell cycle distribution determined 6 hours after serum readdition. For each phase, all groups with different letters are significantly different from each other, *p* < 0.05, n = 3).

Over-expression of recombinant NUDT6 results in perturbation of cell cycle progression [5,19]. We used siRNA knockdown to examine the effect of endogenous NUDT6 and FGF2 on cell cycle progression. Cells were transfected with control or gene-specific siRNAs, then arrested and synchronized in G0/G1 by serum starvation (0.01% FBS for 48 hours) as previously described [19]. As shown in Figure 7a, cells transfected with a negative control siRNA rapidly re-entered the cell cycle following re-addition of 10% FBS, with 16.77 ± 1.3% of control cells in S-phase within 3 hours following serum replacement. The number of control cells in S-phase increased further to more than 20% by 6 hours, and remained at this level for the duration of the experiment. Knock-down of FGF2 resulted in a highly significant decrease in the number of cells entering S-phase, an effect which was detectable as early as 3 hours after serum addition (5.93 ± 0.8% *vs*. 16.77 ± 1.3% in controls), and which persisted for up to 24 hours. Consistent with the cell proliferation results observed in Figure 6, knock-down of NUDT6 also caused a delay in S-phase re-entry, although the effect was not as profound as that seen with FGF2 knockdown (Figure 7a). At 3 hours after serum readdition, the number of cells in S phase was 10.63 ± 1.3% in NUDT6 knockdowns *vs*. 5.93 ± 0.8% in FGF2 knockdowns. The effect of NUDT6 knockdown on S phase reentry persisted for up to 12 hours, but by 24 hours there was no significant difference from controls. 

We next examined the effect of siRNA treatment on cell cycle progression. As shown in Figure 7b, both FGF2 and NUDT6 knockdown resulted in significantly increased accumulation of cells in the G0/G1 phases of the cell cycle, and significant decreases in the population of S-phase cells, consistent with the results observed in Figure 7a. However, NUDT6 knockdown resulted in a significant reduction in the percentage of cells in G2/M (1.33 ± 0.3% *vs*. 3.5 ± 0.4% in controls) whereas FGF2 knockdown resulted in a significant increase in the percentage of cells in G2M (6 ± 0.8%). These data are consistent with our previous report that FGF2 promotes progression from G1 to S phase [19], and further indicate that coordinated expression of both FGF2 and NUDT6 is required for normal cell cycle progression, although at different stages of the cycle. 

### 2.6. Effect of Knockdown on Cell Transformation

**Figure 8:**
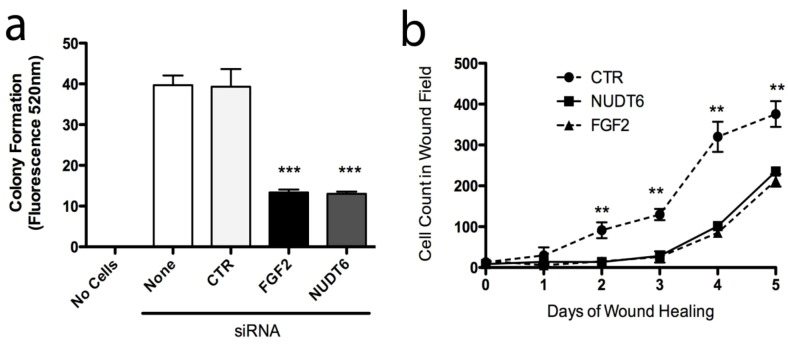
Knockdown of FGF2 or NUDT6 inhibits anchorage-independent growth and wound healing. (**a**) Both FGF2 and NUDT6 siRNA (100 nM) significantly reduced colony formation in soft agar (*** *p* < 0.001 *vs*. transfection with negative control siRNA); (**b**) Inhibition of wound healing activity following siRNA knockdown of FGF2 or NUDT6 (** *p* < 0.01 *vs*. FGF2 or NUDT6 siRNA treatment).

In order to examine the functional implications of FGF2 and NUDT6 expression on C6 glioma cell behaviour, we assessed the effect of knockdown on features of cell behaviour that characterize the neoplastic phenotype, specifically: anchorage-independent growth, adhesion to extracellular matrix proteins, cell migration, invasiveness, and wound healing. Knockdown of either FGF2 or NUDT6 significantly reduced cell adhesion to wells coated with collagen I, collagen IV, and laminin I, but not to fibronectin or fibrinogen (data not shown). Knockdown of either target also significantly inhibited anchorage-independent growth in soft agar by more than 60% compared to controls (Figure 8a). Similarly, knockdown of either FGF2 or NUDT6 caused a highly significantly delay in wound healing over the 5 day duration of the experiment (Figure 8b). There was no significant effect of either knockdown on cell migration or on cell invasiveness as measured using commercially available kits (data not shown). 

Taken together, these findings support the hypothesis that the complementary RNAs encoding FGF2 and NUDT6 are mutually regulatory, and that silencing of either gene results in up-regulation of the corresponding antisense partner, with consequent disruption of cell cycle progression and cell proliferation. 

## 3. Experimental Section

### 3.1. Chemicals and Reagents

Culture medium, fetal bovine serum (FBS) and all other culture reagents were from Gibco BRL (Burlington, Ont., Canada). Gels, membranes and other materials used in Western blotting were, unless otherwise noted, from Bio-Rad Laboratories, Hercules, CA. Anti-FGF2 IgG was obtained from Santa Cruz Biotechnology, Santa Cruz, CA; and anti-NUDT6 and anti-actin were from ProteinTech Group, Chicago, IL, (all rabbit polyclonal). Alexa Fluor^TM^ 488 (green) goat anti-rabbit fluorescent IgG conjugate was from Invitrogen, Carlsbad, CA. All other chemicals were from Sigma-Aldrich Chemical Company, St. Louis, MO, unless otherwise specified.

### 3.2. Cell Culture and Transfection

Rat C6 glioblastoma cells, obtained from the American Type Culture Collection (Manassas, VA, USA), were cultured in Dulbecco’s modified Eagle’s medium (DMEM) supplemented with 10% FBS, penicillin (100 units/mL) and streptomycin (0.1 mg/mL), and kept at 37 °C in a humidified, 5% CO_2 _chamber. Transfection with the specific Silencer^TM^ siRNAs (Applied Biosystems, Austin, TX) was according to the manufacturer’s instructions, with Lipofectamine^TM^ RNAiMAX (Life Technologies Corp., Carlsbad, CA, USA) used as the transfection reagent.

### 3.3. RNA Isolation and RT-PCR Amplification

Total RNA was isolated using the RNeasy^TM^ kit from Qiagen Inc., Mississauga, Ont., Canada, as previously described [17], and reverse-transcribed with Omniscript^TM^ reverse transcriptase (Qiagen). Amplification of the transcripts used 35 cycles of 45 sec at 95 °C, 60 sec at 57 °C and 60 sec at 72 °C with the primers listed in Table 1 (all obtained from Invitrogen), and the HotStar Taq^TM^ master mix (Qiagen). DNA levels were subsequently normalized against the β-actin PCR product amplified from the same RT reaction. 

### 3.4. Protein Extraction and Western Blotting

C6 cells were tranfected as required and incubated for 72 hours. The cells were trypsinized, pelleted at 400 × g for 10 min, washed with phosphate-buffered saline (PBS), and resuspended in RIPA buffer (50 mM Tris, 150 mM NaCl, 50 mM Na_2_HP04, 1 mM Na_3_V0_4_, 1 mM NaF, 5 mM EDTA, 5 mM EGTA, 0.25% Na deoxycholate, 0.1% NP40) supplemented with a protease inhibitor cocktail (Sigma-Aldrich Canada Ltd., Oakville, ON, Canada). Protein was extracted following 30 minutes on ice with frequent vortexing and quantified with a Bradford assay using coomassie reagent (Pierce, Rockford, IL). Equal amounts of protein (30 μg) from each lysate were separated with SDS-PAGE using 12% NuPAGE® Novex® Bis-Tris mini gels in a Xcell SureLock^TM^ Novex Mini-Cell (Life Technologies Corp., Carlsbad, CA, USA) and transferred to a nitrocellulose membrane for western analysis as previously described [16]. Immunodetection of proteins was conducted with fluorescent conjugated secondary antibodies anti-mouse 750 (1:2500) and anti-rabbit 680 (1:5000) (Life Technologies Corp., Carlsbad, CA, USA) against mouse monoclonal FGF-2 (1:200, sc-135905) and rabbit polyclonal Actin (1:1000, sc-1616) (Santa Cruz Biotechnology, Inc., CA, USA). Immunoreactive bands were detected using the LI-COR Biosciences Odyssey^TM^ Imager and quantified using ImageJ.

**Table 1:** Primers used for PCR amplification.

Target	Primer Sequence ^a^, 5' to 3'	Product Size (bp)
*FGF2*	f: GAG GAG TTG TGT CCA TCA AG	230
	r: GGC CTT CTG TCC AGG CCC CG	
*NUDT6*	f: GCT CTT GCA GGC CGC CAT TCA G	226
	r: AAA TAC GGC ACC TGC AAC CCC TA	
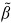	f: TGG CCT TAG GGT TCA GAG GGG	244
	r: ATC GTG GGC CGC CCT AGG CA	

^a ^f = forward, r = reverse.

### 3.5. Immunofluorescent Confocal Laser Scanning Microscopy

For immunofluorescence studies, knockdown transfections were performed in dishes containing microscope coverslips. Following 72 hours incubation, the cells were fixed with ice-cold 2% paraformaldehyde (with 9 mg/mL disodium hydrogen orthophosphate and 6 mg/mL L-lysine) for 15 min, permeabilized with ice-cold 0.1% Triton X-100 in PBS for 15 min and then blocked with 3% bovine serum albumin (BSA) in PBS for one hour (13,18). Staining was by sequential exposure to primary antibodies and their fluorescently tagged secondary antibodies, each for an hour at room temperature, followed by staining for DNA using propidium iodide (PI, 50 ng/mL in PBS) for 10 min. All antibodies were diluted in 0.1% BSA-PBS as follows: anti-FGF2, 2 μg/mL; anti-NUDT6, 3 μg/mL; and Alexa Fluor^TM^ 488 (green) anti-rabbit fluorescent IgG conjugate, 40 μg/mL. Control staining to eliminate antibody non-specificity was performed by application of secondary antibodies without prior exposure of the cells to the primaries, or following incubation with the primary antibody’s preimmunizing peptide. The coverslips were upended onto slides with a drop of glycerol-PBS (Citifluor^TM^, Marivac, Halifax, NS, Canada), and image analysis used the standard operating software on the Zeiss LSM 510 microscope. 

### 3.6. Cell Cycle, FGF2 Staining Intensity and Proliferation Analyses

Cells were plated at a density of 2 × 10^5^ cells/cm^2^ on culture dishes in normal (10% FBS) DMEM and allowed to attach overnight. The cells were then washed with PBS, transfected as appropriate, and incubated for 48 hours in 0.01% FBS DMEM (serum deprivation) to synchronize them in G0. The media were then replaced with normal DMEM and the cells harvested at indicated intervals for DNA content analysis. In brief, the trypsinized cells were centrifuged (400 × g, 10 min at 4 °C), fixed overnight in 70% ethanol at 4 °C and incubated for 30 min at room temperature in PBS with 50 ng/mL propidium iodide and 100 units/mL ribonuclease A [19]. To assess FGF2 content, transfected cells were harvested on successive days, fixed in ethanol, and incubated with an anti-FGF2 antibody−Alexa Fluor secondary antibody combination. Fluorescence intensities were determined in a Becton Dickinson FACSCalibur ^TM^ flow cytometer, the proportion of cells in different phases of the cycle estimated using the ModFit program. Cellular FGF2 immunofluorescence following siRNA treatment was quantified using FCS Express V3 as previously described [19]. Minimum and maximum fluorescence cutoffs were set following background subtraction using cells stained with secondary antibody only. FGF2 immunoflourescence values were determined from the modal fluorescence intensity readings for each treatment. siRNA transfections were performed in triplicate, and three samples from each transfection were used for immunofluorescence readings. Each reading was repeated three times, and the average fluorescence recorded for each sample. 

In the proliferation assay, the cells were grown in low serum (1% FBS) DMEM (control) or 1% FBS-DMEM with 10 ng/mL recombinant human FGF2 (EMD Biosciences), then harvested on successive days and counted through a Coulter counter. 

### 3.7. Cell Transformation Assays

The assessment of cell transformation and tumorigenic tendency was conducted following knockdown transfections and 72 hours incubation of the cells, and all assays were performed using CytoSelect ^TM^ Assay kits (Cell Biolabs, Inc., San Diego, CA, USA) according to the manufacturer’s instructions for each kit: 

#### 3.7.1. Anchorage-Independent Cell Growth

The transfected cells’ ability to form colonies in soft agar was analyzed using the CytoSelect ^TM^ 96-well Cell Transformation Assay kit. Briefly, following knockdown transfection and 72 hours incubation, the cells were seeded into agar-plated wells (75 μL of a 1:1:1 mixture of 1.2% agar solution, 2X DMEM/20% FBS medium, and 4.0 × 10^5^ cells/mL cell suspension for each well). After 15 min incubation at 4 °C to allow the cell-agar layer to solidify, the cells were incubated at 37 °C for six days. The agar was then solubilized, and the cells were lyzed and stained for fluorometric assessment under the PerkinElmer EnVision^TM^ multilabel reader.

#### 3.7.2. Cell Adhesion

The cell adhesion assay was conducted with the CytoSelect ^TM^ 48-well Cell Adhesion Assay kit, as previously described (17). Briefly, the transfected cells were seeded into the assay wells coated with various extracellular matrix proteins (150 μL of a 1.0 × 10^6^ cells/mL suspension per well, in serum-free DMEM) and allowed to attach for 90 min. The cells were then washed and stained, and the stain solubilized for colorimetric assay at 560 nm under the BioTek Instruments PowerWave^TM^ spectrophotomer.

#### 3.7.3. Cell Migration

Cells were analyzed for differences in cell migration using the CytoSelect ^TM^ 24-well Cell Migration Assay, as previously described (17). Briefly, 300 μL of 1.0 × 10^6^ cells/mL serum free suspensions of transfected cells were incubated for 22 hours at 37 °C in polycarbonate membrane inserts with 8 μm pores, surrounding wells containing 10% FBS DMEM. Media and non-adherent cells were then removed from the inserts and migratory cells that had passed through the pores were washed and stained. The cell stain was solubilized, and absorbance was measured at 560 nm.

#### 3.7.4. Cell Invasion

Cells were analyzed for cell invasiveness using the CytoSelect ^TM^ 24-well Cell Invasion Assay, as previously described (17). Briefly, 250 μL of 1.0 × 10^6^ cells/mL serum free cell suspensions were incubated for 24 hours in polycarbonate membrane inserts with 8 μm pores and upper surface type I collagen coating, surrounding wells containing 10% FBS DMEM. Media and non-invasive cells were then removed from the inserts and invasive cells that had passed through the pores were washed and stained. The cell stain was solubilized and absorbance was measured at 560 nm.

#### 3.7.5. Wound Healing

The cells’ ability to repair lesions in the monolayer was assessed using the CytoSelect ^TM^ 24-well Wound Healing Assay. Briefly, the transfected cells were seeded in wells containing removable inserts (500 μL of 1.0 × 10^6^ cell/mL per well, in 10% FBS DMEM). The cells were incubated overnight for monolayer formation around the inserts, which were then removed to leave a “wound” in each well, and fresh medium was added. Images of the “healing” wounds were captured daily for six days, and the images were analyzed for wound closure (number of cells migrating and proliferating in a defined 0.5 mm^2^ zone of the wound field), using the ImageJ program.

### 3.8. Statistical Analysis

All data were compared using the ANOVA with Student-Newman-Keuls procedure, and are presented as mean ± standard error of the mean (SEM). Differences of *p* < 0.05 were considered significant. 

## 4. Conclusions

It has only recently been recognized that a significant fraction of the human genome is transcribed bidirectionally, resulting in the expression of complimentary “sense” and “antisense” RNAs (S/AS) with the potential for mutual regulation [30]. Attention has largely been focused on the role of non-coding antisense RNAs, and knockdown experiments to address the role of endogenous antisense transcripts have yielded inimical results, suggesting that there is no single model to explain antisense-mediated gene regulation [31]. The overlapping FGF2 and NUDT6 genes were among the first S/AS gene pairs to be identified [10], and are unusual in that both transcripts encode evolutionarily conserved protein products with roles in cell proliferation. Our findings demonstrate that expression of both factors is required at different stages of cell cycle progression, and that knockdown of either factor perturbs normal cell proliferation. More importantly, knockdown of either mRNA resulted in a corresponding increase in the complementary partner mRNA, indicating that the normal expression of these factors, and consequent cell cycle progression, is regulated by interaction of the sense and antisense RNAs. These findings may explain our recent observation that perturbations in the relative abundance of FGF2 and NUDT6 mRNAs was a significant prognostic indicator of disease outcome in esophageal adenocarcinoma [15], and suggest the potential for clinically relevant antisense-based therapeutic strategies for modulation of tumor progression. 
